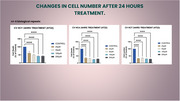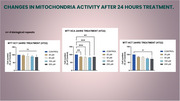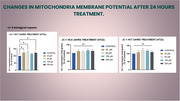# Investigating systems that underpin healthy aging: Examining properties of cells that are surviving neurotoxicity

**DOI:** 10.1002/alz70861_107978

**Published:** 2025-12-23

**Authors:** Oluwajuwonlo Justina Ogungbemi

**Affiliations:** ^1^ University of St Andrews. United Kingdom, St Andrews, Fife UK

## Abstract

**Background:**

Neurodegenerative diseases such as Alzheimer’s disease (AD) are characterised by the progressive loss of specific neuronal populations. However, in every population of neurons that is afflicted in neurodegenerative conditions not all cells die. There remains a subset of survivor neurons. My aim is to elucidate the underlying mechanisms contributing to this differential cellular resilience in neurodegeneration using cell models that are used to model AD such as the hippocampal cell line, HT‐22. One of the known prospective risk factors for AD is elevated levels of the sulphur‐containing amino acid homocysteine (HCY) which is produced as part of the methionine cycle. Its metabolites homocysteine thiolactone (HCT) and homocysteic acid (HCA) are also known to be neurotoxic.

**Method:**

In this study, using a combination of cell viability (crystal violet, lactate dehydrogenase assay, MTT) and biochemical assays, I have modelled neuronal vulnerability *in vitro* using HCY, HCT and HCA as neurotoxins.

**Result:**

My study have shown a decrease in cell number with HCY, HCA and HCT treatment after 24 hours, coupled to an increase in mitochondrial activity in surviving HCY‐treated neurons only, implying differential cellular features of survivor neurons in the HCA and HCT treated cultures. However, there is a decrease in levels of reactive oxygen species generation in the HCA and HCT surviving population suggesting that there may be some antioxidant defenses in place. There was also an increase in NO level in HCA‐treated neurons only which was not seen in HCT.

**Conclusion:**

These preliminary results have established a protocol to further investigate pathways to neuronal vulnerability and resilience in cellular models of AD.